# The Past, Present, and Future of Reproductive Health Surveillance in the US-Mexico Border Region

**Published:** 2008-09-15

**Authors:** Jill A McDonald

**Affiliations:** Centers for Disease Control and Prevention, National Center for Chronic Disease Prevention and Health Promotion, Division of Reproductive Health

This issue of *Preventing Chronic Disease* gives special attention to a recent binational effort to develop novel reproductive health surveillance methods in the US-Mexico border region. The effort began in 2000, when the US and Mexican governments established the United States-Mexico Border Health Commission (USMBHC), specifically to provide international and collaborative leadership to improve health and quality of life in the region (1). The commission created shared US-Mexico health objectives for 2010 related to reproductive health and chronic disease, such as reductions in rates of pregnancy among adolescents and in rates of infant deaths, decreases in rates of diabetes and in rates of cervical and breast cancer mortality, and increases in rates of prenatal care (2).

However, information about factors associated with adverse reproductive health and chronic disease outcomes in this population was not generally available. The Pregnancy Risk Assessment Monitoring System (3), for example, was not designed to collect data representative of the US border population, and no similar system exists in Mexico. Public health officials needed a surveillance system devoted to the special circumstances of the binational border population that could address the information gap surrounding *Healthy Border 2010* health objectives.

The Brownsville-Matamoros Sister City Project for Women's Health (BMSCP) was a demonstration project that explored the feasibility of conducting reproductive health surveillance in the region. Six original articles from the BMSCP are included in this issue. These manuscripts were identified as priority topics by program directors from the collaborating governmental health institutions. McDonald et al describe the surveillance methods that were developed and used to produce the BMSCP data (4). The other 5 manuscripts address topics related to specific *Healthy Border 2010* objectives. Castrucci et al examine the prevalence of breastfeeding initiation, a predictor of infant health (5). Robles et al assess contraceptive use in relation to pregnancy intention, which has implications for family planning services in these communities and for infectious and chronic disease in the population (6). Gossman et al measure levels of prenatal testing for human immunodeficiency virus infection and social and behavioral factors related to such testing (7). Galván González et al estimate fertility among adolescents and young adults and describe their sociobehavioral characteristics (8). Castrucci et al characterize women who ever had a Papanicolaou test and discuss implications for reaching *Healthy Border 2010* goals to reduce cervical cancer mortality (9).

Many health officials, hospital staff, university personnel, and other community members made this work possible. The manuscripts reflect the collaborative spirit in which they collected the data. Mexican and US authors from the Secretariat of Health in Tamaulipas, the Instituto Mexicano del Seguro Social in Tamaulipas, the Texas Department of State Health Services, and the Centers for Disease Control and Prevention (CDC) have participated in each analysis. The analyses present information about each community (Cameron County, Texas, and Matamoros, Tamaulipas) separately, making some comparisons between the 2 communities, and present information on the 2 communities combined.

A lesson from the BMSCP is the critical role that trust and commitment at the local level played in the success of the project. Although the USMBHC set a precedent for the principle of binational collaboration in the region, the practice of working side by side required personal relationships built on commitment to mutual community health goals. Four editorials from the United States and Mexico present additional observations on the project, reflecting state and national perspectives on the BMSCP methods and where they may lead.

Data utility is a key evaluation measure used to assess successful surveillance systems. A determinant in whether decisions will be made to replicate these methods elsewhere in the border region will be the extent to which BMSCP data are used to assess reproductive and other health needs, program reach, and health policy impact in these border communities, as well as to suggest programmatic and political approaches to local health promotion. The United States-Mexico Border Health Association (USMBHA) was responsible for piloting the BMSCP methods and owns the BMSCP data. Data are available for analysis and can be accessed through USMBHA by following guidelines for a mini-proposal process that was established in collaboration with the local institutions and CDC (10).

Replication of BMSCP methods in other sister cities will also depend on the availability of local financial support for surveillance and the capacity to use the data effectively to ask and answer questions about the effectiveness of programs and services, to plan and evaluate interventions, and ultimately to make a difference in maternal, child, and family health. The ability to make comparisons between communities on the border will give us insight into programmatic successes and shortcomings that we can use to make changes within each community. Surveillance data are a critical first step in the process leading to public health action that will help these communities reach health objectives laid out by the USMBHC for 2010 and beyond.
